# Cause of Extra-Uterine Conceptions

**Published:** 1851-09

**Authors:** Silas Hubbard

**Affiliations:** Buffalo


ART. VI.— *Cause of Extra-Uterine Conceptions.* By SILAS HUBBARD, M. D.

SIR,—On page 252, vol. 6, of your Journal, I advanced the theory that males are begotten from one to ten days before, and females from one to ten days after the courses of the mother. In connection with this theory I have, since its publication, also observed, so far as I have ascertained, that extra-uterine fœtations are males. If this is universally true, the questions arise, why are they males, and what are their causes? They may be caused by their being begotten before the menses. The ovum being impregnated before it has made as much progress towards gaining access into the cavity of the uterus, as after the catamenia, is therefore more liable to fail entering the uterus. I have but two cases in proof of this theory: one recorded by Professor Dunglison, on page 441, vol. 2, sixth edition of his Physiology; and one reported by W. H. Watkins, M. D., of Racine, Wisconsin, in the last March No. of your Journal. In cases which would either prove or disprove these theories, writers do not generally state the sex. It would be very useful if they would.

BUFFALO, August, 1851.

